# Mitochondrial Metabolic Checkpoints in Human Fertility: Reactive Oxygen Species as Gatekeepers of Gamete Competence

**DOI:** 10.3390/cells15020149

**Published:** 2026-01-14

**Authors:** Sofoklis Stavros, Nikolaos Thomakos, Efthalia Moustakli, Nikoleta Daponte, Dimos Sioutis, Nikolaos Kathopoulis, Athanasios Zikopoulos, Ismini Anagnostaki, Chrysi Christodoulaki, Themos Grigoriadis, Ekaterini Domali, Anastasios Potiris

**Affiliations:** 1 Third Department of Obstetrics and Gynecology, University General Hospital “ATTIKON”, Medical School, National and Kapodistrian University of Athens, 12462 Athens, Greece; sfstavrou@med.uoa.gr (S.S.); dsioutis@gmail.com (D.S.); 2 First Department of Obstetrics and Gynecology, Alexandra Hospital, Medical School, National and Kapodistrian University of Athens, 11528 Athens, Greece; nthomakos@med.uoa.gr (N.T.); nickatho@gmail.com (N.K.); tgregos@med.uoa.gr (T.G.); kdomali@yahoo.fr (E.D.); 3 Department of Nursing, School of Health Sciences, University of Ioannina, 45500 Ioannina, Greece; 4 Department of Obstetrics and Gynecology, Faculty of Medicine, University of Thessaly, 41110 Larissa, Greece; nikolettadaponte@gmail.com; 5 Torbay and South Devon NHS Foundation Trust Lowes Brg, Torquay TQ2 7AA, UK; thanzik92@gmail.com; 6 Medical School, National and Kapodistrian University of Athens, 11527 Athens, Greece; isanagnostaki3@gmail.com; 7 Department of Obstetrics and Gynecology, General Hospital of Chania, 73300 Chania, Greece; christodoulakichr@hotmail.com

**Keywords:** mitochondria, ROS, gamete competence, OS, oocyte maturation, sperm capacitation, ART

## Abstract

Crucial regulators of gamete metabolism and signaling, mitochondria synchronize energy generation with redox equilibrium and developmental proficiency. Once thought of as hazardous byproducts, reactive oxygen species (ROS) are now understood to be vital signaling molecules that provide a “redox window of competence” that is required for oocyte maturation, sperm capacitation, and early embryo development. This review presents the idea of mitochondrial metabolic checkpoints, which are phases that govern gamete quality and fertilization potential by interacting with cellular signaling, redox balance, and mitochondrial activity. Recent research shows that oocytes may sustain a nearly ROS-free metabolic state by blocking specific respiratory-chain components, highlighting the importance of mitochondrial remodeling in gamete competence. Evidence from in vitro and in vivo studies shows that ROS act as dynamic gatekeepers at critical points in oogenesis, spermatogenesis, fertilization, and early embryogenesis. However, assisted reproductive technologies (ARTs) may inadvertently disrupt this redox–metabolic equilibrium. Potential translational benefits can be obtained via targeted techniques that optimize mitochondrial function, such as modifying oxygen tension, employing mitochondria-directed antioxidants like MitoQ and SS-31, and supplementing with nutraceuticals like melatonin, CoQ10, and resveratrol. Understanding ROS-mediated checkpoints forms the basis for developing biomarkers of gamete competence and precision therapies to improve ART outcomes. By highlighting mitochondria as both metabolic sensors and redox regulators, this review links fundamental mitochondrial biology to clinical reproductive medicine.

## 1. Introduction

Human fertility depends on the coordinated regulation of molecular, cellular, and metabolic pathways that produce gametes capable of fertilization and embryo development [1]. Energy production is a factor which affects gamete quality and is mostly controlled by mitochondria. Beyond their conventional function as “powerhouses”, these dynamic organelles integrate energy metabolism with apoptosis regulation, calcium signaling, and redox balance. Successful reproduction depends on maintaining the integrity of the mitochondria. The ability of oocytes and sperm to mature is directly impacted by the metabolic state of the mitochondria [2,3,4].

Critical to mitochondrial action is the tightly regulated production of reactive oxygen species (ROS). ROS are now recognized to be essential regulators of gamete function, despite previously being believed to be only harmful byproducts of oxidative metabolism [3]. Early embryonic development, sperm capacitation, meiotic oocyte maturation, and the acrosome reaction are all regulated by normal ROS levels. ROS act as molecular “gatekeepers” in these circumstances, coordinating transcriptional networks and intracellular cascades that are essential for reproductive fitness [5].

Elevated or abnormal levels of ROS, which exceed antioxidant capacity, can trigger oxidative stress (OS), mitochondrial dysfunction, and structural or chromosomal damage in gametes. By interfering with cellular signaling, this oxidative damage to macromolecules like proteins, lipids, and DNA eventually lowers embryo quality and fertilization potential and contributes to infertility [6,7,8].

The balance between the generation and elimination of ROS in reproductive cells is mostly regulated by mitochondria. Whether a gamete will fertilize is often determined by the effectiveness of these mitochondrial checkpoints [6]. To control mitochondrial activity, they react to variables like redox balance, oxygen levels, and nutrition use. Mitochondria respond by sending coordinated signals that promote cell integrity. Further influencing gamete maturation and general reproductive capacity are mechanisms such as mitochondrial biogenesis, dynamics, and quality control [9,10].

Recent studies using in vitro and in vivo models show that redox balance and mitochondrial metabolism are highly sensitive to physiological and environmental changes [11]. During oocyte maturation and follicle development, mitochondria adapt to ensure sufficient ATP production and proper redox signaling. Similarly, ROS production and mitochondrial activity are critical for sperm motility and capacitation. Researchers and medical professionals are adopting a more balanced perspective on how oxidative metabolism affects fertility because ROS can both improve and degrade gamete quality [12].

In assisted reproductive technologies (ARTs), it is crucial to distinguish between beneficial and harmful ROS signaling, as mitochondrial and redox biology gain clinical relevance. This review highlights the dual role of ROS in regulating gamete competence and summarizes recent findings on mitochondrial metabolic checkpoints in human fertility. To enhance reproductive outcomes, we also demonstrate how targeted redox modulation can be used as a diagnostic and therapeutic approach, connecting molecular understanding with practical applications. To improve the effectiveness and safety of contemporary reproductive medicines, we conclude by presenting a conceptual framework that connects ROS homeostasis and mitochondrial metabolism.

In this review, we propose the concept of mitochondrial metabolic checkpoints as an integrative framework in reproductive biology. We hypothesize that distinct mitochondrial states, which are characterized by redox signaling, bioenergetic capacity, and mitochondrial DNA (mtDNA) copy number, function as regulatory thresholds influencing gamete competence, fertilization success, and early embryonic development. Rather than acting solely as passive energy producers, mitochondria actively integrate metabolic and redox cues to regulate progression through critical reproductive stages. This framework unifies previously disparate findings and provides a mechanistic basis for the development of mitochondrial biomarkers and targeted interventions in assisted reproductive technologies.

## 2. Methods

This narrative review provides an overview of current research on mitochondrial metabolism and redox control in human fertility, aiming to outline mitochondrial metabolic checkpoints that influence gamete competence and reproductive outcomes in ART. The literature search was performed between January and September 2025 in PubMed, Scopus, and Web of Science using terms such as “oocyte maturation”, “sperm capacitation”, “mitochondria”, “ROS”, “OS”, “gamete competence”, “assisted reproduction”, “redox signaling”, and “fertility”.

The search included in vitro and in vivo studies in humans and animals, as well as clinical and translational research in reproductive biology. Peer-reviewed publications from 2015–2025 were prioritized, with earlier fundamental studies included if conceptually or mechanistically relevant. A selection of the literature addressed ROS dynamics, mitochondrial function, and their impact on the physiology of sperm and oocytes, fertilization, and the development of early embryos.

This paper does not follow a systematic review format but organizes existing literature into thematic groups using an integrative, interpretive approach. The goal was to explore the relationship between redox balance and mitochondrial function and their impact on reproductive capacity. Studies were assessed for methodological rigor, mechanistic insight, and translational relevance. ROS signaling and mitochondrial dynamics are closely linked and are key determinants of gamete quality.

## 3. Mitochondria as Metabolic Regulators of Gamete Competence

The mitochondria are essential organelles for evaluating gametes’ capacity for development. They coordinate energy metabolism, apoptosis, redox signaling, and ATP synthesis as metabolic regulators. These mitochondrial processes, which are collectively known as gamete competence, are necessary for sperm and oocytes to fertilize and maintain embryonic development. During gametogenesis, mitochondrial metabolism and bioenergetics are dynamically reprogrammed to fulfill the evolving requirements of developmental processes [13,14,15].

### 3.1. Mitochondrial Function in the Oocyte

Oocyte mitochondria are crucial for supporting early development because of their unique functional and structural traits. To provide enough energy for the energy-demanding processes of early cleavage, fertilization, and meiosis, oogenesis entails the large-scale production and distribution of mitochondria [16]. Since the metabolic state is quiescent, which lowers the production of ROS during growth, mitochondria are typically spherical, have minimal OXPHOS activity, and undeveloped cristae. Mitochondrial activity rises when the oocyte approaches to metaphase II because spindle construction, chromosomal separation, and cytoplasmic maturation all demand more [17,18].

The quality of oocytes is greatly impacted by the distribution of mitochondria in the ooplasm. A homogeneous cytoplasmic distribution is linked to strong developmental competence, whereas peripheral aggregation or clustering is frequently associated with cytoplasmic immaturity and a limited fertilization potential [19]. Moreover, a crucial bioenergetic and developmental characteristic is the number of copies of mitochondrial DNA (mtDNA). Since mtDNA replication is either low or nonexistent until implantation, substantial mtDNA replication during oogenesis guarantees the formation of a sufficient mitochondrial pool to sustain fertilization and early embryonic development. Inadequate mtDNA copy quantity has been linked to lower implantation potential, cleavage arrest, and poor fertilization outcomes, as well as age-related reproductive decline and impaired oocyte quality. Therefore, mtDNA abundance serves as a quantitative mitochondrial checkpoint that connects eventual embryonic competence with oocyte maturation [20,21].

Processes including mitophagy, fission, and mitochondrial fusion dynamically alter metabolic output and maintain mitochondrial integrity to satisfy developmental demands [22]. While fusion promotes the exchange of contents between mitochondria, diluting harmful chemicals, fission enables damaged mitochondria to be selectively eliminated through mitophagy. Timely oocyte maturation and fertilization help the oocyte adjust to metabolic stress while maintaining mitochondrial health and redox equilibrium [23,24].

### 3.2. Mitochondrial Function in Spermatozoa

Sperm have highly specialized mitochondria crucial for capacitation, motility, and fertilization. The mitochondrial sheath, which is wrapped helically around the axoneme in the midpiece, produces ATP through oxidative phosphorylation. This localized energy supports flagellar movement and the signaling pathways needed for capacitation and the acrosome reaction [25,26].

Although sperm tail glycolysis can also produce ATP, mitochondrial activity is necessary for calcium homeostasis, ROS-mediated signaling, and membrane potential. Important capacitation mechanisms, such as protein tyrosine phosphorylation, hyperactivation, and cholesterol efflux, are triggered by the sperm mitochondria’s controlled production of ROS [27,28]. However, excessive amounts of ROS can affect sperm function and lead to mtDNA damage, motility loss, and lipid peroxidation of the plasma membrane, all characteristic features of male factor infertility [29].

Since sperm mitochondria are transcriptionally silent and unable to go through mitophagy or significant protein synthesis, they are particularly vulnerable to oxidative damage [30]. Additionally, because paternal mitochondria are preferentially destroyed after fertilization, their integrity has a greater impact on fertilization potential than on subsequent embryonic development. Therefore, ROS generation and mitochondrial membrane potential (ΔΨm) are useful indicators of sperm quality and fertilization capacity [31].

### 3.3. Mitochondrial–Nuclear Communication and Metabolic Integration

Constant bidirectional transmission between the nucleus and mitochondria ensures coordination of cellular metabolism and stress response pathways. Nuclear-derived signals (including ROS, ATP/ADP ratio, and NAD^+^/NADH balance) influence nuclear gene expression, while nuclear-encoded transcription factors (such as PGC-1α, NRF1/2, and TFAM) control mitochondrial biogenesis and antioxidant defense. Gametogenic and developmental signals are synchronized with energy consumption through this mitochondrial–nuclear link [32,33,34].

Since transcription is reduced in the later stages of oogenesis and is completely inactive in mature sperm, this communication is particularly important in gametes. Hence, mitochondrial signaling is essential for regulation, linking the cell’s redox status to post-translational changes and epigenetic control. Dysregulation of this interaction results in decreased embryo viability, oocyte aging, and impaired gametogenesis [35].

### 3.4. Mitochondrial Quality Control and Gamete Competence

As a component of mitochondrial quality control, biogenesis, dynamics, and mitophagy cooperate to preserve the integrity of organelles. During gametogenesis, defective mitochondria are selectively eliminated through mitophagy, ensuring that only functional organelles are transmitted to the next generation [36]. Sperm’s mitochondria are highly vulnerable to metabolic or environmental stress because they cannot repair themselves as well as oocytes, which depend on autophagy to remove damaged mitochondria and maintain embryonic competence [37].

Research indicates that one of the main causes of age-related declines in fertility is the deterioration of oocyte mitochondrial quality. Some signs of this decline include decreased ATP synthesis, inadequate calcium regulation, and an increase in ROS accumulation [38]. Lifestyle factors such as smoking, obesity, and exposure to environmental toxins can harm mitochondrial function in gametes. It highlights the importance of maintaining mitochondrial homeostasis as a key predictor of reproductive potential [8]. Table 1 summarizes the main mitochondrial characteristics, functions, and quality-control mechanisms in human gametes, illustrating how these parameters collectively define gamete competence.

## 4. ROS: Dual Roles in Reproductive Physiology

Mitochondrial oxidative metabolism is the main generator of ROS, which are incredibly reactive chemicals present in very small amounts. Although ROS were originally thought to be harmful byproducts that result in cellular death, they are now recognized as crucial signaling intermediates in gamete physiology [49]. Temporal dynamics, location, and concentration all significantly impact their behavior. Physiological levels of ROS mediate significant regulatory mechanisms that support early embryo development and fertilization, as well as gamete maturation. However, when ROS generation exceeds the capacity of antioxidant mechanisms to buffer it, OS results, endangering cellular integrity and fertility [50].

### 4.1. Sources and Regulation of ROS in Gametes

The mitochondria are the main sites where ROS are produced in oocytes and spermatozoa. Superoxide anions (O_2_•^−^) are created when complexes I and III of the electron transport chain (ETC) lose electrons due to oxidative phosphorylation. These radicals are then converted to hydrogen peroxide (H_2_O_2_) by mitochondrial superoxide dismutase (SOD2). After cysteine residues undergo reversible oxidation, H_2_O_2_ can pass across membranes and function as a signaling molecule to change the activity of proteins [51,52].

There are several non-mitochondrial origins of ROS production in gametes. NADPH oxidases (enzymes of the NOX family), cytochrome P450 systems, and xanthine oxidase are examples of localized redox signaling [53]. Sperm capacitation depends on the generation of controlled ROS bursts by plasma membrane-associated NOX5 and mitochondrial ETC activity. Short-term increases in ROS during meiotic restart or fertilization cause oocytes to undergo cell cycle progression and cortical granule exocytosis [40].

The cellular equilibrium of ROS is maintained by a complex network of enzymatic and non-enzymatic antioxidant systems. While glutathione peroxidases (GPx), catalase, and SODs are crucial enzymes, non-enzymatic defenses include coenzyme Q10, vitamins C and E, glutathione (GSH), and melatonin. Depending on the delicate balance between ROS production and antioxidant scavenging, they can either function as physiological messengers or as pathological dangers [54,55].

### 4.2. Physiological Roles of ROS in Reproduction

ROS are essential second messengers that regulate key aspects of gamete physiology and early embryonic development when they are present in moderation. ROS are active signaling molecules that control a variety of processes, including gametogenesis, fertilization, and the early stages of embryogenesis, rather than just being detrimental metabolic byproducts. The physiological effects of ROS necessitate careful temporal and spatial control because even minute variations in ROS set off networks of signaling cascades that promote cellular activity and differentiation [56,57,58].

To perform the acrosome reaction and the capacitation process, ROS are necessary in the sperm’s mitochondrial sheath and plasma membrane. During capacitation, modest concentrations of hydrogen peroxide and superoxide induce cholesterol efflux at the plasma membrane, leading to increased fluidity and reconfiguration of membrane domains [37]. This, in turn, triggers adenylyl cyclase, increasing cAMP and promoting PKA activity. Tyrosine residues are phosphorylated on flagellar and membrane proteins, later increasing motility, hyperactivation, and responsiveness of sperm to acrosome reactions. These well-controlled redox changes are required to achieve fertilization competence and, hence, demonstrate that physiological ROS function as signaling checkpoints rather than simply being injurious agents [27,59].

ROS levels in oocytes temporarily increase during meiotic resumption and cytoplasmic maturation. Controlled oxidation facilitates the transition from prophase I to metaphase II by modifying redox-sensitive proteins implicated in the maturation-promoting factor (MPF) and mitogen-activated protein kinase (MAPK) pathways [60]. Moreover, ROS affect gap-junction communication and cumulus cell activity, both of which are critical for the oocyte’s communication with the surrounding somatic cells and the interchange of metabolites and signals. Furthermore, ROS impacts cumulus cell activity and gap-junction communication, both of which are essential for the exchange of metabolites and signals as well as the oocyte’s connection with the surrounding somatic cells [61,62].

Physiological ROS continue to have significant regulatory functions after fertilization. Oocyte activation and pronuclear production are triggered by ROS spikes in conjunction with calcium oscillations. They also aid in metabolic reprogramming and the modification of gene expression, which helps the early embryo transition from maternal to embryonic genome control [63]. It has been demonstrated that low-level ROS signaling activates pathways related to mitochondrial biogenesis and antioxidant defense, hence sustaining the energy requirements of blastocyst development and cleavage. Early developmental events are guided by metabolic and signaling networks that incorporate ROS throughout this short physiological window [64,65].

Regulated ROS generation is intrinsically associated with reproductive signaling processes. Beyond passive observation, ROS function as active mediators which influence gene transcription, phosphorylation pathways, and ion channel regulation via reversible cysteine oxidation [66]. When sperm and oocytes remain within proper redox levels, redox signaling supports the acquisition and maintenance of the functional states necessary for successful fertilization and early embryonic development [67].

### 4.3. Pathological ROS Excess and OS

OS affects the structure and function of gametes when ROS levels surpass antioxidant capacity. Excess ROS in sperm can oxidize plasma membrane lipids, damage mtDNA, and damage the mitochondrial membrane, which reduces motility, viability, and increases DNA fragmentation. Male infertility and decreased success rates with ART, such as IVF, are caused by these abnormalities [29,68].

OS interferes with ATP synthesis, chromosomal alignment, and spindle formation in oocytes. Long-term exposure to ROS inhibits oocyte development, speeds up ovarian aging, and destroys mitochondrial proteins and DNA. Embryos may stop developing or undergo cell death if oxidant imbalance prevents metabolic reprogramming [46,69].

Aging, obesity, smoking, and exposure to pollutants contribute to increased OS in reproductive cells. Redox homeostasis may be impaired by experimental procedures, including cryopreservation and in vitro culture, that modify oxygen and nutrient availability. Thus, for optimal fertility treatment outcomes and reproductive health, a healthy redox environment must be maintained [70].

### 4.4. The Redox Balance Concept in Gamete Function

In reproductive redox biology, ROS are a double-edged sword. While high ROS damage cells, moderate levels help signaling and cellular regulation. The optimal range for gamete function is referred to as the “redox window of competence”—a state in which ROS concentrations are sufficient to promote signaling but not high enough to induce OS. Within this spectrum, reversible oxidative changes act as regulatory switches that facilitate communication between cells [71].

**Figure 1:**
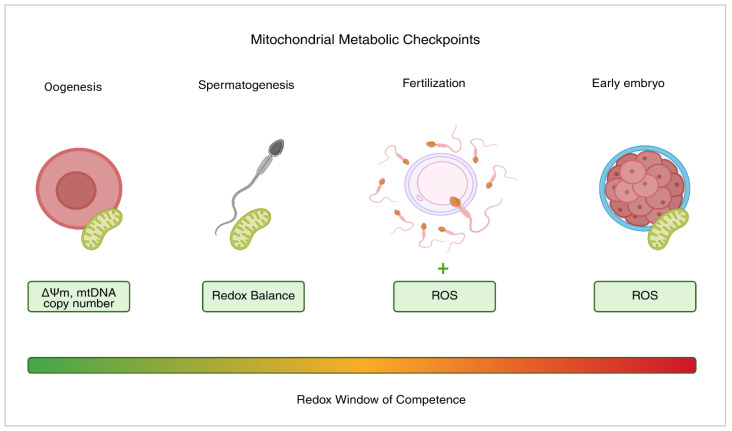
Mitochondrial metabolic checkpoints in human fertility. Schematic representation of the main stages where mitochondrial function and ROS regulate gamete competence and early embryo development. Green zones indicate physiological ROS levels that support signaling and maturation, while red zones represent excess ROS leading to OS and reduced fertility. The plus sign indicates a physiological increase in ROS required for normal fertilization-related signaling.

A stable environment, strong antioxidants, and healthy mitochondria are essential to sustain this balance. When excessive electron leakage damages the mitochondria, the process is interrupted. Conversely, high membrane potential mitochondria generate just enough ROS for signaling without resulting in OS [72]. Table 2 summarizes the physiological versus pathological effects of ROS in human gametes, whereas Figure 1 illustrates how mitochondria integrate energy metabolism and redox signaling to act as metabolic checkpoints during gametogenesis and early embryonic development.

## 5. Mitochondrial Metabolic Checkpoints in Gametogenesis and Fertilization

During key stages of gametogenesis and fertilization, mitochondrial metabolic checkpoints coordinate redox balance, mitochondrial function, and cellular energy management. These checkpoints represent points at which energy production, mitochondrial activity, and ROS signaling converge to regulate gamete maturation. Gametes with adequate metabolic capacity are more likely to effectively navigate through these regulatory checkpoints, while impaired mitochondrial function may limit developmental competence. In this way, mitochondria help safeguard reproductive success by integrating energy production and redox signaling during gametogenesis and fertilization [13,74].

Considered as mitochondrial metabolic checkpoints, this section offers an integrated analysis of mtDNA dynamics, redox signaling, and mitochondrial bioenergetics throughout gametogenesis, fertilization, and early embryonic development.

### 5.1. Oogenesis Checkpoint: Mitochondrial Remodeling and Redox Signaling

Mitochondria proliferate and disperse as oogenesis begins, preparing the oocyte for its increasing energy requirements. Oocytes’ oxidative phosphorylation increases, their mitochondria begin to proliferate, and their inner structure, known as the cristae, grows as they go from their earliest stages to maturity. All of these modifications cause the mitochondria to become most active at specific critical times, which is what actually triggers meiosis and aids in the oocyte’s final maturation [4,75,76].

Rodríguez-Nuevo et al. (2022) [77] provided a major mechanistic advance by demonstrating that human and *Xenopus* oocytes actively suppress complex I of the mitochondrial electron transport chain, thereby maintaining a near-ROS-free metabolic state during prolonged meiotic arrest. This suppression reflects a deliberate metabolic rewiring in which electrons predominantly enter the respiratory chain through complex II and alternative dehydrogenases, rather than resulting from mitochondrial inactivity. This arrangement minimizes electron leakage and superoxide formation while enabling enough ATP production [77]. Oocytes maintain developmental competence throughout prolonged periods of dormancy, minimize oxidative damage to mitochondrial DNA and spindle-associated structures, and maintain mitochondrial integrity by separating oxidative phosphorylation from excessive NADH oxidation at complex I. Crucially, progression toward meiotic restart requires controlled reactivation of mitochondrial ROS signaling, as this metabolic configuration establishes a redox-defined mitochondrial threshold rather than indiscriminate oxidative metabolism. These results emphasize complex I modulation as a context-dependent mechanism that allows oocytes to balance bioenergetic sufficiency with long-term redox protection, even though emerging data from mouse models suggest that complex I activity may not be entirely suppressed in all mammalian species [78].

Since it initiates spindle assembly and meiosis, a small amount of regulated ROS generation during this period is actually crucial. Mild OS promotes healthy chromosome alignment, promotes organelle remodeling, and activates the MPF and MAPK pathways [60]. However, these functions are disrupted by high levels of ROS, which can negatively impact spindle organization, chromosomal alignment, calcium homeostasis, mitochondrial membrane potential (ΔΨm), and cell viability. Redox balance modulation during mitochondrial remodeling is therefore a crucial metabolic checkpoint that directly affects the ability to fertilize oocytes, rather than a secondary effect [60]. Collectively, these findings indicate that mitochondrial metabolic checkpoints in oogenesis are dynamic rather than fixed, adapting to developmental timing and species-specific reproductive strategies.

In this context, complex I modulation emerges as a defining feature of the oocyte mitochondrial metabolic checkpoint, linking redox quiescence to long-term preservation of gamete competence.

### 5.2. Spermatogenesis Checkpoint: Mitochondrial Maturation and ROS Modulation

In male gametogenesis, tightly regulated phases of sperm maturation are dominated by redox signaling and mitochondrial differentiation [17]. The midpiece is formed by the migration and helical wrapping of mitochondria around the axoneme during spermatid elongation, which supplies a localized energy source for motility. This rearrangement improves the efficiency of oxidative phosphorylation by raising the amounts of ATP synthase and cytochrome c oxidase [79]. In spermatogenesis, physiological ROS serve as crucial signaling molecules that facilitate transcriptional and post-translational mechanisms that encourage sperm differentiation and capacitation. Antioxidant enzymes, such as superoxide dismutase and peroxiredoxins, help maintain balance [80]. Nevertheless, oxidative damage can build up in mitochondrial membranes and mtDNA when ROS generation surpasses antioxidant capacity, resulting in immature sperm, morphological abnormalities, and decreased motility [37].

### 5.3. Fertilization Checkpoint: Mitochondrial Cross-Talk and ROS Signaling

Mitochondrial signaling is thus an intrinsic part of the gametes’ successful fusion and activation during fertilization, which involves a great metabolic shift [81]. The short bursts of ROS generated by mitochondria in oocytes upon repeated calcium oscillations trigger cortical granule exocytosis, therefore helping to prevent polyspermy. Such ROS-mediated pathways are also important in pronuclear formation and in the oocyte activation process; hence, a well-balanced oxidative environment must be maintained during fertilization [82].

Paternal mitochondria contribute to early ROS signaling but are selectively removed post-fertilization via ubiquitin-dependent mitophagy, ensuring maternal-only mitochondrial inheritance. Therefore, the mitochondrial population from the oocyte to a large extent dictates the redox state of the zygote [15].

ATP synthesis, mitochondrial membrane potential, and mtDNA integrity are necessary for the fertilized oocyte to control the metabolic shift into cleavage and early embryogenesis [83]. The fusion of once distinct metabolic pathways is symbolized by the mitochondrial checkpoint, which is created during fertilization by modifying both gametes. Through the balance of energy generation and redox signaling, this integration promotes early embryonic growth [13].

### 5.4. Early Embryo Development Checkpoint: Redox Control of Zygotic Competence

After fertilization, the early embryo undergoes multiple cleavage divisions and significant metabolic reprogramming [84]. Rapid cell proliferation drives a shift from pyruvate-dependent oxidative metabolism to glycolysis, while the immature mitochondria, with sparse cristae, help limit ROS overproduction. However, the production of transitory ROS is an important signaling mechanism involved in lineage specification, cell cycle regulation, and embryonic genome activation [85].

A redox imbalance or disruption of mitochondrial activity during this stage may hinder development or compromise the likelihood of implantation. Studies have demonstrated that in ART, embryos with optimal mitochondrial activity and balanced ROS levels had higher implantation rates and better developmental outcomes [86]. Therefore, maintaining mitochondrial metabolic integrity and redox equilibrium during early cleavage is the final checkpoint influencing embryo survival. Thus, tightly regulated bioenergetics and redox signaling enable mitochondrial metabolic checkpoints to contribute to the regulation of gamete competence and early embryonic viability [87].

In early embryogenesis, mtDNA copy number represents an essential mitochondrial checkpoint that extends beyond redox signaling. Before mitochondrial biogenesis resumes, a minimum threshold of mtDNA content is necessary to support ATP synthesis throughout the cleavage phases. Reduced implantation potential, delayed development, and poor metabolic flexibility are characteristic of embryos derived from oocytes with insufficient mtDNA copy number. To combine oocyte mitochondrial quality with embryonic viability and reproductive success, mtDNA copy quantity acts as a developmental gatekeeper [88]. Table 3 summarizes the key mitochondrial activities, ROS functions, and consequences of imbalance across oogenesis, spermatogenesis, fertilization, and early embryo development.

## 6. Mitochondrial Redox Regulation in Assisted Reproduction

For the mitochondria of gametes, ART is a special form of ex vivo stress test. Various steps in the procedure, such as oocyte retrieval, sperm selection, and embryo culture, are known to expose cells to mechanical stress, nonphysiological oxygen tension, and altered nutrient availability [17,73]. Each of these may cause a shift in the redox balance and interfere with mitochondrial metabolic checkpoints that normally maintain gamete competence. Clinically, the efficiency of such checkpoints is reflected in the success of ART, wherein redox disturbances at any of the oocyte maturation, sperm handling, fertilization, or embryo culture steps impact mitochondrial function either positively or negatively [92].

### 6.1. Redox Homeostasis During Oocyte Retrieval and In Vitro Maturation

Oocyte retrieval and in vitro maturation (IVM) are highly sensitive to oxidative conditions. Excess ROS may be produced in the oocytes and surrounding cumulus cells if ambient oxygen levels (about 20%) are exposed instead of the natural ovarian environment (approximately 5%). Elevated ROS levels are known to reduce intracellular ATP, disturb the meiotic spindle, and compromise mitochondrial membrane potential, all of which can impair oocyte quality [93].

Lowering oxygen tension during IVM has been shown to improve developmental outcomes. Under near-physiological oxygen conditions, antioxidant enzymes remain active, mitochondria stay functional, and oocytes are better able to complete maturation [94]. To further enhance spindle integrity, maintain mitochondrial health, and maintain redox balance, antioxidants like melatonin, resveratrol, or CoQ10 can be added to the maturation medium. It will ultimately increase the possibility for fertilization [95].

More recently, mitochondria-targeted antioxidants like MitoQ and SkQ1 have attracted interest [96]. These compounds accumulate within the inner mitochondrial membrane, the main site of ROS generation, where they appear to support cytoplasmic maturation and reduce mitochondrial depolarization under OS. However, time and concentration significantly influence their actions; therefore, careful optimization is required to maximize benefits while minimizing unintended harm [97].

### 6.2. Sperm Mitochondrial Function in Assisted Fertilization

Centrifugation, swim-up, and density-gradient separation are sperm preparation techniques that may inadvertently increase ROS production by removing seminal plasma antioxidants [98]. OS may result from ROS accumulation in the absence of this protective environment. High levels of ROS can harm mitochondrial DNA, interfere with the electron transport chain, and hinder sperm movement, all of which lower the success rate of fertilization [99].

As biomarkers for sperm quality, mitochondrial functional assays, such as measures of mitochondrial superoxide levels, ATP content, and membrane potential (ΔΨm), are increasingly being used in ART clinics. Centrifugation and the elimination of seminal plasma antioxidants can interfere with mitochondrial redox checkpoints during sperm production [8,100]. Newer methods, including microfluidic sperm sorting, maintain the ideal ROS signaling needed for capacitation and better protect mitochondrial integrity. These tests might be added to standard diagnostic methods to improve the selection of metabolically competent spermatozoa, which would speed up the development of embryos and fertilization [101].

### 6.3. Embryo Culture and Oxidative Microenvironment

Due to their immature antioxidant systems, in vitro-developing embryos are particularly vulnerable to redox imbalance. Abnormal gene expression, impaired ATP synthesis, and changes to mtDNA can be caused by excess ROS generated by extended culture, excessive oxygen exposure, or light irradiation [50]. Low-oxygen culture methods (5% O_2_) more closely mimic physiological tubal settings by maintaining mitochondrial activity within the optimal range. Furthermore, by adding modest antioxidants (such as cysteine, β-mercaptoethanol, or vitamin E equivalents) to embryo media, oxidative damage can be prevented without preventing the natural ROS signaling required for embryo genome activation [102].

Mitochondrial activity has recently been suggested as a possible indicator of embryo viability. Non-invasive methods such as detecting mitochondrial-derived metabolites or cell-free mtDNA in spent culture media may offer crucial insights into the embryo’s health prior to implantation. These mitochondrial indications may someday supplement, or even surpass, conventional morphological grading methods [103,104].

### 6.4. Therapeutic Targeting of Mitochondrial Dysfunction

Growing evidence of the link between mitochondrial function and reproductive health is driving new therapeutic approaches. For example, oocytes with severe mitochondrial problems may benefit from mitochondrial replacement therapy (MRT), though ethical and regulatory concerns remain [21]. Gentler alternatives are also being explored, such as boosting NAD^+^ with compounds like nicotinamide riboside or enhancing mitochondrial biogenesis via PGC-1α pathways [105].

A balanced environment is provided by ART for the evaluation and improvement of mitochondrial function. ART cycles may benefit from targeted redox techniques to address metabolic abnormalities, and mitochondrial function is further supported by a balanced diet, vitamin intake, and lifestyle choices [106]. Regular exercise and nutraceuticals such as omega-3 fatty acids and CoQ10 may assist in restoring equilibrium, regenerating mitochondria, and strengthening antioxidant defenses [107].

Treatment plans and laboratory procedures can be rethought when ART is viewed through the lens of mitochondrial metabolism. Personalized ART is made possible by the direct correlation between mitochondrial health and reproductive results through the use of real-time mitochondrial indicators to inform decision-making [108].

Heterogeneous research designs, inconsistent dosing schedules, and a lack of long-term safety data continue to hinder the clinical translation of mitochondria-targeted medicines despite promising preclinical findings [109]. Crucially, redox-modulating therapies often show biphasic or dose-dependent effects, whereby excessive antioxidant supplementation may inhibit physiological ROS signaling that is necessary for the development of gametes and embryos. Recent evidence highlights the need for precise dose optimization and timing, as long-term or high-dose exposure to compounds such as melatonin may alter oocyte epigenetic programming. Therefore, rather than being standardized therapies in ART, mitochondria-targeted medicines should now be considered experimental or adjunctive techniques [86,110].

## 7. Potential Biomarkers and Therapeutic Strategies

Mitochondria are essential for the proper function of gametes, like sperm and oocytes. According to recent studies, gamete quality can be determined by specific mitochondrial characteristics [41]. For instance, cells that have fewer mtDNA copies tend to develop less efficiently. ATP synthesis and general mitochondrial function depend on the mitochondrial membrane potential (ΔΨm); disturbances in this potential might impair oocyte quality and sperm motility. In summary, these cellular energy manufacturers play a major role in gamete fertility [111].

Although ROS and RNS play essential roles in cell signaling, their excess can damage DNA and impair cellular function. The antioxidant defense mechanisms of gametes involve enzymatic components including glutathione peroxidase, catalase, and superoxide dismutase [99]. By protecting cells from OS and maintaining structural integrity, these molecules complement ongoing research into non-invasive methods for real-time monitoring of living cells. An indication of a cell’s energy condition, mitochondrial activity, and developmental potential is its composition of metabolites in the culture medium. Lipid peroxides and oxidized glutathione are two examples of OS indicators which can be detected in the surrounding media [112].

Several mitochondria-targeted approaches are under investigation to improve gamete mitochondrial health; however, their clinical efficacy and safety remain incompletely defined. Melatonin, resveratrol, and coenzyme Q10 (CoQ10) exhibit antioxidant properties and have been reported to stabilize mitochondrial membrane potential and promote mitochondrial biogenesis in experimental settings [113,114]. Melatonin may have mitochondrial protective effects through both antioxidant and receptor-mediated mechanisms, whereas resveratrol has been demonstrated to activate sirtuin-dependent pathways involved in cellular energy control. As a crucial part of the electron transport chain, CoQ10 may increase mitochondrial efficiency and prevent age-related reductions in mitochondrial function [115].

However, there is still little and inconsistent evidence of consistent improvements in human fertility results. Crucially, redox-modulating treatments often exhibit dose-dependent or biphasic effects, whereby excessive antioxidant supplementation may inhibit physiological ROS signaling required for normal gamete maturation and embryonic development [116]. Recent studies indicate that high-dose or prolonged exposure to certain antioxidants, including melatonin, can affect oocyte epigenetic regulation, underscoring the importance of rigorous dose optimization and long-term safety evaluation. Similar to this, drugs that target mitochondria, including MitoQ and SS-31 (elamipretide), have demonstrated encouraging results in animal and in vitro trials by enhancing mitochondrial activity and lowering oxidative damage in gametes; however, solid clinical data in humans are still absent [117,118].

Improved gamete quality and decreased oxidative stress have been linked to lifestyle and nutritional changes that support mitochondrial and metabolic health, such as regular exercise, a balanced diet, and quitting smoking [119,120]. Collectively, available data indicate that mitochondria-targeted therapies remain experimental or adjunctive approaches in assisted reproduction and require validation through carefully designed, adequately powered clinical trials that address patient selection, dosing strategies, and long-term reproductive safety.

## 8. Challenges and Future Perspectives

There is still much to understand about how mitochondria function and how cells regulate redox balance in gametes before any of this can be applied in actual reproductive clinics. The lack of a standardized method for measuring ROS or other indicators of OS in labs is one of the major issues [38]. Comparing findings across studies is challenging because researchers often use different methodologies. Therefore, a key priority is to establish accurate and reliable methods for detecting OS in gametes and early embryos [121].

Another component is that redox biology and standard fertility tests must be integrated. Although ROS were once assumed to be harmful, we now know that they play an important role in signaling for early embryo development, gamete maturation, and fertilization. In light of this, healthcare professionals might assess the quality of oocytes and sperm much more accurately if we could develop instruments that distinguish between “normal” and “trouble” levels of ROS [122]. Furthermore, multi-omics approached offer significant promise. Researchers can begin deciphering the intricate connections among ROS signaling, gamete quality, and mitochondrial function by integrating transcriptomics, metabolomics, and other large-scale data methodologies. Reliance on a single type of analysis may overlook novel biomarkers or potential therapeutic targets [123].

The precise levels of ROS that support optimal gamete function remain unclear. Although the mechanisms and timing are not yet fully understood, both excessive and insufficient OS have been shown to impair fertility. More careful observation of mitochondrial and redox changes is required before firm recommendations can be made for ART [124]. To address these challenges, laboratories should standardize testing protocols and integrate redox biology into routine diagnostic procedures. The impact of mitochondrial networks and ROS on gamete quality can then be better understood by using multi-omics techniques. This could transform fertility care by facilitating customized treatments and better prediction of gamete and embryo success [110].

## 9. Conclusions

Mitochondria are essential for successful reproduction because they integrate signaling and energy metabolism, influencing gamete competence, fertilization, and early embryonic development. In this study, we present a model that connects mitochondrial function with key metabolic and redox pathways in reproductive cells.

Several mitochondrial metabolic markers have been identified as indicators of gamete quality, linking redox signaling to energy production. Through this framework, we illustrate how ROS serve as molecular checkpoints that bridge redox biology and reproductive science. Importantly, maintaining the appropriate “redox window”, rather than simply minimizing ROS, appears to be critical for optimal fertility.

By providing direction for the development of pharmaceutical treatments, dietary supplements, and culture systems that improve mitochondrial function while maintaining redox balance, this viewpoint can help to inform ART. To monitor genuine progress, future research should adopt standardized redox biomarkers, high-resolution metabolic imaging, and multi-omics approaches. These resources can help reproductive medicine go from empirical approaches to tailored treatments based on a thorough molecular understanding of mitochondrial function.

## Figures and Tables

**Table 1:** This table provides an overview of the traits, roles, and quality control systems of the mitochondria in human gametes.

Feature/Function	Oocyte Mitochondria	Sperm Mitochondria	Functional Relevance to Gamete Competence
Localization/Structure [39,40]	Evenly distributed throughout cytoplasm; spherical, sparse cristae	Helically arranged around axoneme in midpiece; dense cristae	Proper distribution ensures uniform ATP supply and signaling efficiency
Bioenergetic Role [41]	ATP generation for meiosis, spindle formation, fertilization, and early embryo development	ATP production for motility, capacitation, and acrosome reaction	Sustains energy-intensive processes essential for fertilization
mtDNA Copy Number [8,42]	High (~100,000–500,000 copies); replicated during oogenesis	Low (~50–100 copies); no replication post-spermatogenesis	Determines embryo developmental capacity; low mtDNA linked to poor oocyte quality
Mitochondrial Dynamics [43]	Active fusion/fission cycles; mitophagy maintains quality	Limited dynamics; minimal mitophagy or biogenesis after maturation	Dynamic turnover preserves functional mitochondria in oocytes; sperm rely on pre-formed organelles
Redox Regulation/ROS Generation [4]	Low basal ROS; increases during maturation and fertilization	Moderate ROS for capacitation; excessive ROS impairs motility and DNA integrity	Controlled ROS supports signaling; imbalance causes oxidative damage
Calcium Homeostasis [44,45]	Buffers cytoplasmic Ca^2+^ fluctuations during activation	Regulates Ca^2+^ needed for motility and acrosome reaction	Couples energy metabolism with signaling cascades
Mitochondrial–Nuclear Crosstalk [46,47]	Strong communication via PGC-1α, NRF1/2, TFAM; influences epigenetic programming	Minimal transcriptional feedback; relies on pre-synthesized proteins	Coordinates energy metabolism and gene expression during gametogenesis
Quality-Control Mechanisms [8,46]	Mitophagy removes damaged organelles; rejuvenation during oogenesis	Lacks significant repair; defective mitochondria accumulate with age/environmental stress	Maintains mitochondrial integrity and developmental potential
Age and Environmental Sensitivity [48]	Highly sensitive to maternal age, metabolic and OS	Susceptible to temperature, toxins, and lifestyle factors	Mitochondrial dysfunction contributes to reduced fertility in both sexes

**Table 2:** This table summarizes the physiological versus pathological effects of ROS in human gametes.

ROS Level/Context	Biological Role	Molecular Mechanisms	Outcome on Gamete Competence
Low/Controlled (Physiological) [12]	Signaling mediator	Activation of protein kinases (PKA, MAPK); reversible oxidation of cysteine residues; modulation of ion channels and transcription factors	Promotes sperm capacitation, oocyte maturation, and early embryonic development
Moderate (Adaptive Stress) [73]	Preconditioning response	Upregulation of antioxidant enzymes (SOD, GPx); mitochondrial biogenesis via PGC-1α activation	Enhances stress resilience and maintains gamete functionality
High/Chronic (Pathological) [6]	OS	Lipid peroxidation, mtDNA damage, protein carbonylation, apoptosis	Impairs sperm motility, oocyte quality, fertilization rate, and embryo viability
Environmental/ART-induced [8]	Secondary oxidative imbalance	Exposure to high O_2_ tension, cryopreservation, culture media imbalance	Reduced gamete competence, increased embryonic fragmentation

**Table 3:** Mitochondrial metabolic checkpoints in gametogenesis and early development.

Checkpoint/Stage	Key Mitochondrial Activities	ROS Role	Consequences of Imbalance
Oogenesis [4]	Mitochondrial biogenesis, redistribution, cristae elaboration, oxidative phosphorylation	Moderate ROS signals meiosis onset and spindle assembly	Excess ROS → spindle abnormalities, chromosomal misalignment, apoptosis, impaired calcium homeostasis
Spermatogenesis [89,90]	Mitochondrial maturation, axoneme wrapping, increased ATP synthase and cytochrome c oxidase	Physiological ROS modulates transcription, post-translational processes, sperm differentiation	Excess ROS → mtDNA and membrane damage, sperm immaturity, morphological defects, reduced motility
Fertilization [91]	Mitochondrial cross-talk, ΔΨm maintenance, ATP production, mtDNA integrity	Transient ROS bursts mediate calcium oscillations, cortical granule exocytosis, pronuclear formation	ROS imbalance → impaired gamete activation, failed fertilization; improper paternal mitochondria removal can affect zygotic redox tone
Early Embryo Development [8,92]	Metabolic reprogramming (pyruvate → glycolysis), immature mitochondrial cristae, ATP production	Transient ROS regulates cell cycle, lineage specification, embryonic genome activation	Redox or mitochondrial dysfunction → hindered cleavage, reduced implantation, compromised embryo survival

## Data Availability

No new data were created or analyzed in this study.

## References

[1] Siu K.K., Serrão V.H.B., Ziyyat A., Lee J.E. (2021). The cell biology of fertilization: Gamete attachment and fusion. J. Cell Biol..

[2] Peng J., Ramatchandirin B., Pearah A., Maheshwari A., He L. (2022). Development and Functions of Mitochondria in Early Life. Newborn.

[3] Chianese R., Pierantoni R. (2021). Mitochondrial Reactive Oxygen Species (ROS) Production Alters Sperm Quality. Antioxidants.

[4] Gałęska E., Kowalczyk A., Wrzecińska M., García M.C., Czerniawska-Piątkowska E., Gwoździewicz S., Witkiewicz W., Dobrzański Z. (2025). The Importance of Mitochondrial Processes in the Maturation and Acquisition of Competences of Oocytes and Embryo Culture. Int. J. Mol. Sci..

[5] Yuan S., Zhang Y., Dong P.Y., Chen Yan Y.M., Liu J., Zhang B.Q., Chen M.M., Zhang S.E., Zhang X.F. (2024). A comprehensive review on potential role of selenium, selenoproteins and selenium nanoparticles in male fertility. Heliyon.

[6] Song J., Xiao L., Zhang Z., Wang Y., Kouis P., Rasmussen L.J., Dai F. (2024). Effects of reactive oxygen species and mitochondrial dysfunction on reproductive aging. Front. Cell Dev. Biol..

[7] Potiris A., Moustakli E., Trismpioti E., Drakaki E., Mavrogianni D., Matsas A., Zikopoulos A., Sfakianakis A., Tsakiridis I., Dagklis T. (2025). From Inflammation to Infertility: How Oxidative Stress and Infections Disrupt Male Reproductive Health. Metabolites.

[8] Vahedi Raad M., Firouzabadi A.M., Tofighi Niaki M., Henkel R., Fesahat F. (2024). The impact of mitochondrial impairments on sperm function and male fertility: A systematic review. Reprod. Biol. Endocrinol..

[9] Ye L., Fu X., Li Q. (2025). Mitochondrial Quality Control in Health and Disease. MedComm.

[10] Piantadosi C.A., Suliman H.B. (2012). Redox regulation of mitochondrial biogenesis. Free Radic. Biol. Med..

[11] Chen S., Li Q., Shi H., Li F., Duan Y., Guo Q. (2024). New insights into the role of mitochondrial dynamics in oxidative stress-induced diseases. Biomed. Pharmacother..

[12] Henkel R. (2024). Role of mitochondria and redox state in sperm and oocyte health. Gynecol. Reprod. Endocrinol. Metab..

[13] Kankanam Gamage S.U., Morimoto Y. (2025). Significance of Mitochondrial Dynamics in Reproductive Physiology: Current and Emerging Horizons in Mitochondrial Therapy for Assisted Reproductive Technologies. Reprod. Med. Biol..

[14] Elías-López A.L., Vázquez-Mena O., Sferruzzi-Perri A.N. (2023). Mitochondrial dysfunction in the offspring of obese mothers and it’s transmission through damaged oocyte mitochondria: Integration of mechanisms. Biochim. Biophys. Acta BBA-Mol. Basis Dis..

[15] Podolak A., Woclawek-Potocka I., Lukaszuk K. (2022). The Role of Mitochondria in Human Fertility and Early Embryo Development: What Can We Learn for Clinical Application of Assessing and Improving Mitochondrial DNA?. Cells.

[16] Yildirim R.M., Seli E. (2024). The role of mitochondrial dynamics in oocyte and early embryo development. Semin. Cell Dev. Biol..

[17] Almansa-Ordonez A., Bellido R., Vassena R., Barragan M., Zambelli F. (2020). Oxidative Stress in Reproduction: A Mitochondrial Perspective. Biology.

[18] Chiaratti M.R. (2021). Uncovering the important role of mitochondrial dynamics in oogenesis: Impact on fertility and metabolic disorder transmission. Biophys. Rev..

[19] Bahety D., Böke E., Rodríguez-Nuevo A. (2024). Mitochondrial morphology, distribution and activity during oocyte development. Trends Endocrinol. Metab..

[20] Rahimi Darehbagh R., Khalafi B., Allahveisi A., Habiby M. (2022). Effects of The Mitochondrial Genome on Germ Cell Fertility: A Review of The Literature. Int. J. Fertil. Steril..

[21] Podolak A., Liss J., Kiewisz J., Pukszta S., Cybulska C., Rychlowski M., Lukaszuk A., Jakiel G., Lukaszuk K. (2022). Mitochondrial DNA Copy Number in Cleavage Stage Human Embryos-Impact on Infertility Outcome. Curr. Issues Mol. Biol..

[22] Wang S., Tan J., Miao Y., Zhang Q. (2022). Mitochondrial Dynamics, Mitophagy, and Mitochondria-Endoplasmic Reticulum Contact Sites Crosstalk Under Hypoxia. Front. Cell Dev. Biol..

[23] Adebayo M., Singh S., Singh A.P., Dasgupta S. (2021). Mitochondrial fusion and fission: The fine-tune balance for cellular homeostasis. FASEB J..

[24] Youle R.J., Van Der Bliek A.M. (2012). Mitochondrial Fission, Fusion, and Stress. Science.

[25] Park Y.J., Pang M.G. (2021). Mitochondrial Functionality in Male Fertility: From Spermatogenesis to Fertilization. Antioxidants.

[26] Foutouhi A., Meyers S. (2022). Comparative oxidative metabolism in mammalian sperm. Anim. Reprod. Sci..

[27] Takei G.L. (2024). Molecular mechanisms of mammalian sperm capacitation, and its regulation by sodium-dependent secondary active transporters. Reprod. Med. Biol..

[28] Ritagliati C., Baro Graf C., Stival C., Krapf D. (2018). Regulation mechanisms and implications of sperm membrane hyperpolarization. Mech. Dev..

[29] Sengupta P., Pinggera G.M., Calogero A.E., Agarwal A. (2024). Oxidative stress affects sperm health and fertility-Time to apply facts learned at the bench to help the patient: Lessons for busy clinicians. Reprod. Med. Biol..

[30] Durairajanayagam D., Singh D., Agarwal A., Henkel R. (2021). Causes and consequences of sperm mitochondrial dysfunction. Andrologia.

[31] Xu Z., Yan Q., Zhang K., Lei Y., Zhou C., Ren T., Gao N., Wen F., Li X. (2025). Mitochondrial Regulation of Spermatozoa Function: Metabolism, Oxidative Stress and Therapeutic Insights. Animals.

[32] Cagin U., Enriquez J.A. (2015). The complex crosstalk between mitochondria and the nucleus: What goes in between?. Int. J. Biochem. Cell Biol..

[33] Zhang X., Gao Y., Zhang S., Wang Y., Pei X., Chen Y., Zhang J., Zhang Y., Du Y., Hao S. (2025). Mitochondrial dysfunction in the regulation of aging and aging-related diseases. Cell Commun. Signal..

[34] Yong C.Q.Y., Tang B.L. (2018). A Mitochondrial Encoded Messenger at the Nucleus. Cells.

[35] Dvoran M., Nemcova L., Kalous J. (2022). An Interplay between Epigenetics and Translation in Oocyte Maturation and Embryo Development: Assisted Reproduction Perspective. Biomedicines.

[36] Liu B.H., Xu C.Z., Liu Y., Lu Z.L., Fu T.L., Li G.R., Deng Y., Luo G.-Q., Ding S., Li N. (2024). Mitochondrial quality control in human health and disease. Mil. Med. Res..

[37] Wang Y., Fu X., Li H. (2025). Mechanisms of oxidative stress-induced sperm dysfunction. Front. Endocrinol..

[38] Moustakli E., Grigoriadis T., Stavros S., Potiris A., Zikopoulos A., Gerede A., Tsimpoukis I., Papageorgiou C., Louis K., Domali E. (2025). Artificial Intelligence in Assessing Reproductive Aging: Role of Mitochondria, Oxidative Stress, and Telomere Biology. Diagnostics.

[39] Van Blerkom J. (2011). Mitochondrial function in the human oocyte and embryo and their role in developmental competence. Mitochondrion.

[40] Irigoyen P., Pintos-Polasky P., Rosa-Villagran L., Skowronek M.F., Cassina A., Sapiro R. (2022). Mitochondrial metabolism determines the functional status of human sperm and correlates with semen parameters. Front. Cell Dev. Biol..

[41] Phan O.T.H., Trinh T.T.C., Nguyen V.T.T. (2025). Mitochondria in the reproduction system and mitotherapy in assisted reproductive technology: The importance of mitochondria selection. Biomed. Res. Ther..

[42] Nguyen H.T., Do S.Q., Wakai T., Funahashi H. (2025). Mitochondrial content and mtDNA copy number in spermatozoa and penetrability into oocytes. Theriogenology.

[43] Zhao S., Heng N., Wang H., Wang H., Zhang H., Gong J., Hu Z., Zhu H. (2022). Mitofusins: From mitochondria to fertility. Cell. Mol. Life Sci..

[44] Garriga F., Codina-Benaiges J., Yeste M., Llavanera M. (2026). Calcium homeostasis role in preserving sperm function and metabolic activity during liquid storage of pig semen. Theriogenology.

[45] Han Y., Du Z., Wu H., Zhao R., Liu J., Gao S., Zeng S. (2025). CALB1 and RPL23 Are Essential for Maintaining Oocyte Quality and Function During Aging. Aging Cell.

[46] Ju W., Zhao Y., Yu Y., Zhao S., Xiang S., Lian F. (2024). Mechanisms of mitochondrial dysfunction in ovarian aging and potential interventions. Front. Endocrinol..

[47] Zhang W., Wu F. (2023). Effects of adverse fertility-related factors on mitochondrial DNA in the oocyte: A comprehensive review. Reprod. Biol. Endocrinol..

[48] Smits M.A.J., Schomakers B.V., Van Weeghel M., Wever E.J.M., Wüst R.C.I., Dijk F., Janssens G.E., Goddijn M., Mastenbroek S., Houtkooper R.H. (2023). Human ovarian aging is characterized by oxidative damage and mitochondrial dysfunction. Hum. Reprod..

[49] Lindsay R.T., Rhodes C.J. (2025). Reactive Oxygen Species (ROS) in Metabolic Disease—Don’t Shoot the Metabolic Messenger. Int. J. Mol. Sci..

[50] Agarwal A., Maldonado Rosas I., Anagnostopoulou C., Cannarella R., Boitrelle F., Munoz L.V., Finelli R., Durairajanayagam D., Henkel R., Saleh R. (2022). Oxidative Stress and Assisted Reproduction: A Comprehensive Review of Its Pathophysiological Role and Strategies for Optimizing Embryo Culture Environment. Antioxidants.

[51] Zhao R.Z., Jiang S., Zhang L., Yu Z.B. (2019). Mitochondrial electron transport chain, ROS generation and uncoupling (Review). Int. J. Mol. Med..

[52] Wong H.S., Dighe P.A., Mezera V., Monternier P.A., Brand M.D. (2017). Production of superoxide and hydrogen peroxide from specific mitochondrial sites under different bioenergetic conditions. J. Biol. Chem..

[53] de Almeida A.J.P.O., de Oliveira J.C.P.L., da Silva Pontes L.V., de Souza Júnior J.F., Gonçalves T.A.F., Dantas S.H., Feitosa M.S.d.A., Silva A.O., de Medeiros I.A. (2022). ROS: Basic Concepts, Sources, Cellular Signaling, and its Implications in Aging Pathways. Oxidative Med. Cell. Longev..

[54] Jomova K., Raptova R., Alomar S.Y., Alwasel S.H., Nepovimova E., Kuca K., Valko M. (2023). Reactive oxygen species, toxicity, oxidative stress, and antioxidants: Chronic diseases and aging. Arch. Toxicol..

[55] Manful C.F., Fordjour E., Subramaniam D., Sey A.A., Abbey Lord Thomas R. (2025). Antioxidants and Reactive Oxygen Species: Shaping Human Health and Disease Outcomes. Int. J. Mol. Sci..

[56] Lodde V., Morandini P., Costa A., Murgia I., Ezquer I. (2021). cROStalk for Life: Uncovering ROS Signaling in Plants and Animal Systems, from Gametogenesis to Early Embryonic Development. Genes.

[57] Hardy M.L.M., Day M.L., Morris M.B. (2021). Redox Regulation and Oxidative Stress in Mammalian Oocytes and Embryos Developed In Vivo and In Vitro. Int. J. Environ. Res. Public Health.

[58] Hong Y., Boiti A., Vallone D., Foulkes N.S. (2024). Reactive Oxygen Species Signaling and Oxidative Stress: Transcriptional Regulation and Evolution. Antioxidants.

[59] Jin S.K., Yang W.X. (2017). Factors and pathways involved in capacitation: How are they regulated?. Oncotarget.

[60] Voros C., Athanasiou D., Papapanagiotou I., Mavrogianni D., Varthaliti A., Bananis K., Athanasiou A., Athanasiou A., Papadimas G., Gkirgkinoudis A. (2025). Cracking the Code of Oocyte Quality: The Oxidative Stress Link to IVF Success. Int. J. Mol. Sci..

[61] Turathum B., Gao E.M., Chian R.C. (2021). The Function of Cumulus Cells in Oocyte Growth and Maturation and in Subsequent Ovulation and Fertilization. Cells.

[62] Del Bianco D., Gentile R., Sallicandro L., Biagini A., Quellari P.T., Gliozheni E., Sabbatini P., Ragonese F., Malvasi A., D’amato A. (2024). Electro-Metabolic Coupling of Cumulus–Oocyte Complex. Int. J. Mol. Sci..

[63] Mauchart P., Vass R.A., Nagy B., Sulyok E., Bódis J., Kovács K. (2023). Oxidative Stress in Assisted Reproductive Techniques, with a Focus on an Underestimated Risk Factor. Curr. Issues Mol. Biol..

[64] Liu S., Liu J., Wang Y., Deng F., Deng Z. (2025). Oxidative Stress: Signaling Pathways, Biological Functions, and Disease. MedComm.

[65] May-Panloup P., Boguenet M., El Hachem H., Bouet P.E., Reynier P. (2021). Embryo and Its Mitochondria. Antioxidants.

[66] Li B., Ming H., Qin S., Nice E.C., Dong J., Du Z., Huang C. (2025). Redox regulation: Mechanisms, biology and therapeutic targets in diseases. Signal Transduct. Target. Ther..

[67] Onochie C., Evi K., O’Flaherty C. (2025). Role of Redox-Induced Protein Modifications in Spermatozoa in Health and Disease. Antioxidants.

[68] Moustakli E., Zikopoulos A., Katopodis P., Dafopoulos S., Paraschos V.S., Zachariou A., Dafopoulos K. (2025). Dietary and Lifestyle Interventions to Mitigate Oxidative Stress in Male and Female Fertility: Practical Insights for Infertility Management—A Narrative Review. Metabolites.

[69] Sasaki H., Hamatani T., Kamijo S., Iwai M., Kobanawa M., Ogawa S., Miyado K., Tanaka M. (2019). Impact of Oxidative Stress on Age-Associated Decline in Oocyte Developmental Competence. Front. Endocrinol..

[70] Yang H., Kuhn C., Kolben T., Ma Z., Lin P., Mahner S., Jeschke U., von Schönfeldt V. (2020). Early Life Oxidative Stress and Long-Lasting Cardiovascular Effects on Offspring Conceived by Assisted Reproductive Technologies: A Review. Int. J. Mol. Sci..

[71] Chen Y.S., Tian H.X., Rong D.C., Wang L., Chen S., Zeng J., Xu H., Mei J., Wang L.-Y., Liou Y.-L. (2025). ROS homeostasis in cell fate, pathophysiology, and therapeutic interventions. Mol. Biomed..

[72] Khan T., Waseem R., Zehra Z., Aiman A., Bhardwaj P., Ansari J., Hassan I., Islam A. (2022). Mitochondrial Dysfunction: Pathophysiology and Mitochondria-Targeted Drug Delivery Approaches. Pharmaceutics.

[73] Ferreira F.C., Teixeira J., Lidon F., Cagide F., Borges F., Pereira R.M.L.N. (2025). Assisted Reproduction Technologies (ART): Impact of Mitochondrial (Dys)function and Antioxidant Therapy. Animals.

[74] Kang M.H., Kim Y.J., Lee J.H. (2023). Mitochondria in reproduction. Clin. Exp. Reprod. Med..

[75] Urbisz A.Z., Chajec Ł., Małota K., Student S., Sawadro M.K., Śliwińska M.A., Świątek P. (2022). All for one: Changes in mitochondrial morphology and activity during syncytial oogenesis. Biol. Reprod..

[76] Qi L., Chen X., Wang J., Lv B., Zhang J., Ni B., Xue Z. (2019). Mitochondria: The panacea to improve oocyte quality?. Ann. Transl. Med..

[77] Rodríguez-Nuevo A., Torres-Sanchez A., Duran J.M., De Guirior C., Martínez-Zamora M.A., Böke E. (2022). Oocytes maintain ROS-free mitochondrial metabolism by suppressing complex I. Nature.

[78] Ru Y., Deng X., Chen J., Zhang L., Xu Z., Lv Q., Long S., Huang Z., Kong M., Guo J. (2024). Maternal age enhances purifying selection on pathogenic mutations in complex I genes of mammalian mtDNA. Nat. Aging.

[79] Skowronek M.F., Pietroroia S., de Cola G., Ramos M., Silvera D., Casanova G., Lecumberry F., Cassina A., Sapiro R. (2025). Mitochondrial morphology in fertile and infertile men: Image processing and morphometric analysis of the sperm midpiece. Front. Cell Dev. Biol..

[80] Antinozzi C., Di Luigi L., Sireno L., Caporossi D., Dimauro I., Sgrò P. (2025). Protective Role of Physical Activity and Antioxidant Systems During Spermatogenesis. Biomolecules.

[81] Varuzhanyan G., Rojansky R., Sweredoski M.J., Graham R.L.J., Hess S., Ladinsky M.S., Chan D.C. (2019). Mitochondrial fusion is required for spermatogonial differentiation and meiosis. eLife.

[82] Voros C., Mavrogianni D., Athanasiou D., Sapantzoglou I., Bananis K., Athanasiou A., Athanasiou A., Papadimas G., Tsimpoukelis C., Papapanagiotou I. (2025). Rescuing Fertilization Failure in ICSI: A Narrative Review of Calcium Ionophore Activation, PLCζ Testing, and Embryo Morphokinetics. Biomedicines.

[83] Wang T., Xu P., Yuan J., Chen H., Guo X., Gao J., Wang Y., Yao D., Li X., Liu B. (2025). Mitochondrial dysfunction in oocytes: Implications for fertility and ageing. J. Ovarian Res..

[84] Mu J., Zhou Z., Sang Q., Wang L. (2022). The physiological and pathological mechanisms of early embryonic development. Fundam. Res..

[85] Antico Arciuch V.G., Elguero M.E., Poderoso J.J., Carreras M.C. (2012). Mitochondrial regulation of cell cycle and proliferation. Antioxid. Redox Signal..

[86] Divvela S.S.K., Gallorini M., Gellisch M., Patel G.D., Saso L., Brand-Saberi B. (2025). Navigating redox imbalance: The role of oxidative stress in embryonic development and long-term health outcomes. Front. Cell Dev. Biol..

[87] Van Blerkom J., Gardner D.K., Sakkas D., Seli E., Wells D. (2013). Mitochondrial activity as a biomarker of gamete and embryo health. Human Gametes and Preimplantation Embryos.

[88] Winstanley Y.E., Liu J., Adhikari D., Gonzalez M.B., Russell D.L., Carroll J., Robker R.L. (2024). Dynamics of Mitochondrial DNA Copy Number and Membrane Potential in Mouse Pre-Implantation Embryos: Responses to Diverse Types of Oxidative Stress. Genes.

[89] Vedelek V., Jankovics F., Zádori J., Sinka R. (2024). Mitochondrial Differentiation during Spermatogenesis: Lessons from Drosophila melanogaster. Int. J. Mol. Sci..

[90] Juárez-Rojas L., Casillas F., López A., Betancourt M., Ommati M.M., Retana-Márquez S. (2022). Physiological role of reactive oxygen species in testis and epididymal spermatozoa. Andrologia.

[91] Deluao J.C., Winstanley Y., Robker R.L., Pacella-Ince L., Gonzalez M.B., McPherson N.O. (2022). OXIDATIVE STRESS AND REPRODUCTIVE FUNCTION: Reactive oxygen species in the mammalian pre-implantation embryo. Reproduction.

[92] Kobayashi H., Yoshimoto C., Matsubara S., Shigetomi H., Imanaka S. (2024). Altered Energy Metabolism, Mitochondrial Dysfunction, and Redox Imbalance Influencing Reproductive Performance in Granulosa Cells and Oocyte During Aging. Reprod. Sci..

[93] Begum I.A. (2025). Oxidative stress: Oocyte quality and infertility. Reprod. Toxicol..

[94] Rakha S.I., Elmetwally M.A., El-Sheikh Ali H., Balboula A., Mahmoud A.M., Zaabel S.M. (2022). Importance of Antioxidant Supplementation during In Vitro Maturation of Mammalian Oocytes. Vet. Sci..

[95] Rodríguez-Varela C., Labarta E. (2020). Clinical Application of Antioxidants to Improve Human Oocyte Mitochondrial Function: A Review. Antioxidants.

[96] Fields M., Marcuzzi A., Gonelli A., Celeghini C., Maximova N., Rimondi E. (2023). Mitochondria-Targeted Antioxidants, an Innovative Class of Antioxidant Compounds for Neurodegenerative Diseases: Perspectives and Limitations. Int. J. Mol. Sci..

[97] Zong Y., Li H., Liao P., Chen L., Pan Y., Zheng Y., Zhang C., Liu D., Zheng M., Gao J. (2024). Mitochondrial dysfunction: Mechanisms and advances in therapy. Signal Transduct. Target. Ther..

[98] Fleming S., Morroll D., Nijs M. (2025). Sperm Separation and Selection Techniques to Mitigate Sperm DNA Damage. Life.

[99] Wróblewski M., Wróblewska W., Sobiesiak M. (2024). The Role of Selected Elements in Oxidative Stress Protection: Key to Healthy Fertility and Reproduction. Int. J. Mol. Sci..

[100] Krzyściak W., Papież M., Bąk E., Morava E., Krzyściak P., Ligęzka A., Gniadek A., Vyhouskaya P., Janeczko J. (2020). Sperm Antioxidant Biomarkers and Their Correlation with Clinical Condition and Lifestyle with Regard to Male Reproductive Potential. J. Clin. Med..

[101] Pinto S., Carrageta D.F., Alves M.G., Rocha A., Agarwal A., Barros A., Oliveira P.F. (2021). Sperm selection strategies and their impact on assisted reproductive technology outcomes. Andrologia.

[102] Ma Y.Y., Chen H.W., Tzeng C.R. (2017). Low oxygen tension increases mitochondrial membrane potential and enhances expression of antioxidant genes and implantation protein of mouse blastocyst cultured in vitro. J. Ovarian Res..

[103] Kobayashi M., Kobayashi J., Shirasuna K., Iwata H. (2020). Abundance of cell-free mitochondrial DNA in spent culture medium associated with morphokinetics and blastocyst collapse of expanded blastocysts. Reprod. Med. Biol..

[104] Toporcerová S., Badovská Z., Kriváková E., Mikulová V., Mareková M., Altmäe S., Rabajdová M. (2025). Embryo secretome in predicting embryo quality and IVF treatment outcome. Reprod. Biomed. Online.

[105] Yusri K., Jose S., Vermeulen K.S., Tan T.C.M., Sorrentino V. (2025). The role of NAD+ metabolism and its modulation of mitochondria in aging and disease. npj Metab. Health Dis..

[106] Casanova A., Wevers A., Navarro-Ledesma S., Pruimboom L. (2023). Mitochondria: It is all about energy. Front. Physiol..

[107] Drobnic F., Lizarraga M.A., Caballero-García A., Cordova A. (2022). Coenzyme Q10 Supplementation and Its Impact on Exercise and Sport Performance in Humans: A Recovery or a Performance-Enhancing Molecule?. Nutrients.

[108] Cecchino G.N., Seli E., Alves Da Motta E.L., García-Velasco J.A. (2018). The role of mitochondrial activity in female fertility and assisted reproductive technologies: Overview and current insights. Reprod. Biomed. Online.

[109] Gropman A.L., Uittenbogaard M.N., Chiaramello A.E. (2024). Challenges and opportunities to bridge translational to clinical research for personalized mitochondrial medicine. Neurother. J. Am. Soc. Exp. Neurother..

[110] Moustakli E., Christopoulos P., Potiris A., Zikopoulos A., Matsas A., Arkoulis I., Mavrogianni D., Drakaki E., Zachariou A., Drakakis P. (2025). Reductive stress and the role of antioxidants in male infertility: A narrative review. Arch. Gynecol. Obstet..

[111] Picard M., Shirihai O.S. (2022). Mitochondrial signal transduction. Cell Metab..

[112] Anselme M., He H., Lai C., Luo W., Zhong S. (2025). Targeting mitochondrial transporters and metabolic reprogramming for disease treatment. J. Transl. Med..

[113] Rodríguez-Varela C., Labarta E. (2021). Does Coenzyme Q10 Supplementation Improve Human Oocyte Quality?. Int. J. Mol. Sci..

[114] Wen H., Deng H., Li B., Chen J., Zhu J., Zhang X., Yoshida S., Zhou Y. (2025). Mitochondrial diseases: From molecular mechanisms to therapeutic advances. Signal Transduct. Target. Ther..

[115] DiNicolantonio J.J., McCarty M.F., O’Keefe J.H. (2022). Coenzyme Q10 deficiency can be expected to compromise Sirt1 activity. Open Heart.

[116] Moustakli E., Zikopoulos A., Skentou C., Katopodis P., Domali E., Potiris A., Stavros S., Zachariou A. (2024). Impact of Reductive Stress on Human Infertility: Underlying Mechanisms and Perspectives. Int. J. Mol. Sci..

[117] Amaral H.B.S., Silveira M.M., Nicolás A.C.C.V., Pimenta L.K.L., Chaves J.E.V., Caetano A.R., Franco M.M., Dode M.A.N. (2025). Melatonin Improves Bovine Embryo Production and Quality via Antioxidant, Metabolic, and Epigenetic Pathways. Antioxidants.

[118] Qu J., Cai W., Lu Y., Pu D., Tan R., Wu J. (2025). Melatonin mitigates polystyrene nanoplastics-induced impairment of oocyte maturation in mice. Ecotoxicol. Environ. Saf..

[119] Nguyen S.T., Otoi T., Namula Z., Widodo O.S., Tharasanit T., Chatdarong K., Nakayama Y., Nagahara M., Nakai A., Hirata M. (2025). Mitochondrial-Targeted Protective Potential of Elamipretide for the In Vitro Production of Porcine Embryos. Animals.

[120] Grosser J.A., Fehrman R.L., Keefe D., Redmon M., Nickells R.W. (2021). The effects of a mitochondrial targeted peptide (elamipretide/SS31) on BAX recruitment and activation during apoptosis. BMC Res. Notes.

[121] Musson R., Gąsior Ł., Bisogno S., Ptak G.E. (2022). DNA damage in preimplantation embryos and gametes: Specification, clinical relevance and repair strategies. Hum. Reprod. Update.

[122] Tsunoda S., Kimura N., Fujii J. (2014). Oxidative stress and redox regulation of gametogenesis, fertilization, and embryonic development. Reprod. Med. Biol..

[123] Sanches P.H.G., de Melo N.C., Porcari A.M., de Carvalho L.M. (2024). Integrating Molecular Perspectives: Strategies for Comprehensive Multi-Omics Integrative Data Analysis and Machine Learning Applications in Transcriptomics, Proteomics, and Metabolomics. Biology.

[124] Shi Y.Q., Zhu X.T., Zhang S.N., Ma Y.F., Han Y.H., Jiang Y., Zhang Y.H. (2023). Premature ovarian insufficiency: A review on the role of oxidative stress and the application of antioxidants. Front. Endocrinol..

